# The efficiency of nano-TiO_2_ and γ-Al_2_O_3_ in copper removal from aqueous solution by characterization and adsorption study

**DOI:** 10.1038/s41598-021-98051-3

**Published:** 2021-09-22

**Authors:** Fatemeh Ezati, Ebrahim Sepehr, Fatemeh Ahmadi

**Affiliations:** Department of Soil Science, Faculty of Agriculture, Urmia University, Urmia, Iran

**Keywords:** Environmental sciences, Natural hazards, Nanoscience and technology

## Abstract

Water pollution is a major global challenge given the increasing growth in the industry and the human population. The present study aims to investigate the efficiency of TiO_2_ and γ-Al_2_O_3_ nanoadsorbents for removal of copper (Cu(II)) from aqueous solution as influenced by different chemical factors including pH, initial concentration, background electrolyte and, ionic strength. The batch adsorption experiment was performed according to standard experimental methods. Various isotherm models (Freundlich, Langmuir, Temkin, and Dubinin–Radushkevich) were fitted to the equilibrium data. According to geochemical modeling data, adsorption was a predominant mechanism for Cu(II) removal from aqueous solution. Calculated isotherm equations parameters were evidence of the physical adsorption mechanism of Cu(II) onto the surface of the nanoparticles. The Freundlich adsorption isotherm model could well fit the experimental equilibrium data at different pH values. The maximum monolayer adsorption capacity of TiO_2_ and γ-Al_2_O_3_ nanosorbents were found to 9288 and 3607 mg kg^−1^ at the highest pH value (pH 8) and the highest initial Cu(II) concentration (80 mg L^−1^) respectively. Copper )Cu(II) (removal efficiency with TiO_2_ and γ-Al_2_O_3_ nanoparticles increased by increasing pH. Copper )Cu(II) (adsorption deceased by increasing ionic strength. The maximum Cu(II) adsorption (4510 mg kg^−1^) with TiO_2_ nanoparticles was found at 0.01 M ionic strength in the presence of NaCl. Thermodynamic calculations show the adsorption of Cu(II) ions onto the nanoparticles was spontaneous in nature. Titanium oxide (TiO_2_) nanosorbents could, therefore, serve as an efficient and low-cost nanomaterial for the remediation of Cu(II) ions polluted aqueous solutions.

## Introduction

A series of serious environmental problems caused by heavy metals pollution have already pay attention around the world^1^. Heavy metals, being recalcitrant and persistent, can be harmful to humans, animals, plants, and urban ecosystems^2^. Among various heavy metals, copper (Cu(II)) is an essential and vital dietary micronutrient and also found in enzymes where it facilitates the absorption of iron (Fe) and helps to transmit electrical signals in the body^3^. In high doses, however, Cu(II) can be extremely toxic in the human system, resulting to cases like hair loss, hypoglycemia, kidney damage, gastrointestinal problems and even death^4^. High Cu(II) level in the human liver has been reported to cause Wilson’s disease, thalassemia, hemochromatosis, yellow atrophy of liver, tuberculosis and carcinomas^5^. According to the United States Environmental Protection Agency (EPA) and The World Health Organization (WHO), the permissible levels for Cu(II) in drinking water are 1.3 and 2 mg dm^-3^, respectively^2^.

Copper (Cu(II)) is a widely used industrial metal whose applications include electrical wiring, plumbing, air conditioning tubing, fertilizer industry and roofing^6^. High concentration levels of Cu(II) contamination in the industrial wastewaters must be reduced to acceptable levels before discharging them into the environment^7^. Therefore, several studies have been focused on the reduction of Cu(II) entry to the soil, surface water, and human food chain subsequently from various industries notably^8^.

Various physical and chemical methods (such as solvent extraction^9^, membrane filtration^10^, and chemical precipitation^11^) are applied widely for elimination of heavy metals in soil and water. But, most of them are require high costs and may be ineffective at metal concentrations less than 10 mg dm^-3^, etc.^4^. Among all chemical methods, the adsorption process using different nanomaterials is a simple, effective, and low cost technology for remediation of polluted soil and water^12^. Compared with conventional particles, nano-sized particles (such as iron, titanium, and alumina nanoparticles^13^), inevitably have a larger specific surface area which improves their superior adsorption properties^10^. Previous researches showed the high efficiency of hexagonal Fe-based MIL-88B nanocrystals in oxytetracycline adsorption^14^. Van Tran et al.^15^ reported that the Fe_3_O_4_@Cnanocomposite was effective for removing a wide range of organic dyes from aqueous solutions. The efficiency of Zeolitic-imidazolate porous carbons on tetracycline and ciprofloxacin was demonstrated in previous researches^16^. High consistency, appropriate dielectric properties and photocatalytic activity of TiO_2_ lead to acceptable performance of this nanoparticle as an adsorbent^17^, also, γ-Al_2_O_3_ nanoparticles has potentially high resistance to chemical factors, so the nanoparticles are capable of acting as a catalyst in different chemical reactions^18^.

The adsorption process of heavy metals ions over different surfaces is controlled by various factors including the initial ion concentration, the temperature, the contact time, the adsorbent dosage, and the pH of reaction medium. In the study^19^ of the adsorption of Cu(II) ions over the surface of Fe_3_O_4_/SiO_2_/PAN nanocomposite, the adsorption was increased when sodium chloride concentration in the solution was 0.025 mM that enhances the dispersion of functional groups on the adsorbent surfaces. Nanofibrous adsorbent PVA/ZnO adsorption capacity was improved for the chelation of Ni(II), Cu(II), and U(VI) when the metallic initial concentration increased from 90 to 500 mg L^−1^ ^20^. In a study^21^ of Cu(II) ions removal via nano-SiO_2_, there was an increase in the adsorption capacity when the pH changed from 2 to 5. However, what actually happens in the adsorption process is more complex than examining the effect of a single chemical factor, so that several factors may be involved in the ion adsorption process by the adsorbent simultaneously, on the other hand, the adsorption behavior of each ion as affected by factors is different based on the different adsorbents characteristics^22^.

Although the influence of single chemical factor (such as pH, ionic strength, adsorbent dosage, and etc.) on Cu(II) adsorption by TiO_2_, Al_2_O_3_, and other nanosorbents was studied separately in previous researches^23^, but simultaneous assessment of several factors affecting adsorption (such as pH, initial concentration, background electrolyte type, and ionic strength), especially by TiO_2_ and γ-Al_2_O_3_ nanoparticles, as two effective Cu(II) sorbents based on previous researches, has not been investigated so far. Owing to the limitations of studies for removal of Cu(II) species as affected by different chemical factors simultaneously from the aqueous solution, this study examined the simultaneous influence of pH (ranging from 4 to 8), initial ion concentration (maximum 80 mg L^−1^), background electrolyte type (CaCl_2_ and NaCl), and ionic strength (0.01, 0.1, and 0.5 M) on Cu(II) removal from aqueous solution by using titanium and γ-aluminium oxides (TiO_2_ and γ-Al_2_O_3_) nanoparticles using adsorption studies.

## Materials and methods

### Adsorbent

Nanostructured TiO_2_ and γ-Al_2_O_3_ were analytical reagent grade and employed without any further impurity (purity, 99%) from Nanopars Lima (www.Nanopars Lima co, Iran). The morphology of the adsorbents was analyzed by scanning electron microscopy (SEM, S4800, Hitachi) coupled by energy X-ray dispersive (EDX)^24^. All scanning electron microscopy (SEM) images were recorded on a Hitachi S-4800 field-emission SEM microscope (Hitachi Ltd., Chiyoda, Tokyo, Japan). Transmission electron microscopy (TEM) analysis was carried out using a transmission electron microscope (model JEOL 2100F) operated at an accelerating voltage of 200 keV. The surface area and the average pore diameter of the adsorbents were determined using a Micro metrics ASAP 2010 gas adsorption surface analyzer at 77 K (Quantachrome Nova 2000e, USA)^17^. X-ray diffraction (XRD) was used for investigation the structure of the TiO_2_ and γ-Al_2_O_3_ nanoparticles using a X’Pert PRO MPD X-ray diffractometer (Panalytical, Almelo, Netherlands) equipped with Cu Kα radiation (λ = 1.5406 A^o^) (40 kV, 40 mA) at scan rate at 3 s/step.

### Speciation

The speciation is a critical factor affects metal reactivity, including its solubility, adsorption, and precipitation behavior^5^. Determining of metal speciation helps to better understand metal behavior in soil and water^24^. A geochemical speciation model MINTEQ (Visual MINTEQ 3.1, KTH, Sweden; Stockholm) was used to calculate the various Cu species frequency and saturation indices in the aqueous solution with the highest Cu concentration (80 mg L^−1^). Visual MINTEQ is a geochemical equilibrium model extensively used for the accurate calculation of metal speciation, precipitation and solubility of dissolved mineral phase in aqueous solution^4^. The saturation index (SI) is calculated from the difference between the logarithm of the ion activity product (log IAP) and, the logarithm of the temperature corrected solubility constant (log K_s_) for each solid compound of the experimentn^25^. Over-saturation, under-saturation and equilibrium conditions with the solid phase are occurred when SI > 0, SI < 0, and SI = 0 (or more accurately, −0.5 < SI < 0.5) respectively^25^.

### Adsorption isotherms

Batch sorption experiment was performed at room temperature (25 ± 1 °C) in conical flasks by stirring a mass of 0.05 g nanoadsorbents (TiO_2_ and γ-Al_2_O_3_ separately) with 5 mL of Cu(II) solutions at different initial concentrations (0, 2.5, 5, 10, 20, 40, and 80 mg L^−1^) for 2 h at 1000 rpm and left for 4 h at room temperature for equilibration. In order to separate of solid–liquid phases of nanoparticle suspensions, the solutions were centrifuged for 30 min (1000 rpm) and filtered through No. 42 Whatman filter paper^26^. The filtrate was analyzed by Flame Atomic Absorption Spectrophotometer (AAS, Shimadzu AA-6300, Japan) at a wavelength of 325 nm. These initial concentrations were selected based on a concentration range frequently existed in contaminated waters^27^. The stock solution of Cu(II) was prepared by dissolving CuSO_4_·5H_2_O (Merck Co.) in deionized water to the concentration of 1 g L^−1^. The experiment solutions were prepared by diluting the Cu(II) stock solution in accurate proportions to needed initial concentrations^28^. The background solution was 0.01 M calcium chloride (CaCl_2_) to the neutralization of ionic strength^2^.

The effect of pH on the Cu(II) adsorption was studied over the pH range from 6.0 to 8.0 with γ-Al_2_O_3_ and 4.0 to 8.0 with TiO_2_ nanoparticles. The pH of Cu(II) solution was adjusted by using either 0.1 M HCl or 0.1 M NaOH. Each experiment was duplicated under identical conditions with less than 5% standard deviation. The adsorption capacity of the adsorbents at equilibrium was calculated by the following equation^17^:

1 $${Q}_{e}=\left({C}_{0}- {C}_{e} \right)\times V/m$$

where *Q*_*e*_ is the amount of metal ion adsorbed (mg kg^−1^), *C*_*0*_ and *C*_*e*_ (mg L^−1^) are the initial and equilibrium concentration of metal ion solution, respectively. *V* is the volume of adsorbate in liter and *m* is the amount of adsorbent in grams. The formula for calculating the removal efficiency of the adsorbents was expressed by Eq. (2)^29^:

2 $${\text{Removal Efficiency}}\left(\mathrm{\%}\right)=({C}_{0}- {C}_{e})/{C}_{0} \times 100$$

where *C*_*0*_ (mg L^−1^) and *C*_*e*_ (mg L^−1^) are the initial and equilibrium Cu(II) concentrations respectively.

Equilibrium data are basic requirements to understand the mechanism of the adsorption. Classical adsorption isotherm models, Langmuir, Freundlich, Temkin, and Dubinin–Radushkevich (D–R), are used to describe the equilibrium between adsorbed Cu(II) on the adsorbents (q_e_) and Cu(II) concentration in solution (C_e_) at a constant temperature^11^.

The Langmuir adsorption isotherm assumes that adsorption occurs at specific homogeneous sites within the adsorbent and has found successful application in many monolayer adsorption process^30^. The non-linear form of the Langmuir isotherm equation is computed by using the following expression^31^:

3 $${Q}_{m}=({K}_{L}\times {C}_{e} \times {S}_{m})/ (1+{K}_{L}\times {C}_{e})$$

where *Q*_*m*_ and *C*_*e*_ have the same definitions as in Eq. (3), *K*_*L*_ is a constant of the Langmuir equation, that is related to adsorption affiliation of binding sites for ion adsorption (L g^−1^), and *S*_*m*_ is the maximum adsorption capacity with monolayer coverage (mg kg^−1^)^32^.

The effect of isotherm shape has been discussed to predict whether an adsorption system is favorable or unfavorable^3^. The essential feature of the Langmuir isotherm can be expressed by means of ‘*R*_*L*_’, a dimensionless constant referred to as separation factor or equilibrium parameter *R*_*L*_ is calculated using the following equation^18^:

4 $${R}_{L}=\frac{1}{1+ {K}_{L} {C}_{0}}$$

where *K*_*L*_ is the Langmuir constant (dm^3^ mol^−1^) and *C*_*0*_ the highest initial Cu(II) concentration (mol dm^-3^). The dimensionless Langmuir constant, or equilibrium parameter, (*R*_*L*_) indicates if the isotherm is irreversible (*R*_*L*_ = 0), favorable (0 < *R*_*L*_ < 1), linear (*R*_*L*_ = 1), and unfavorable (*R*_*L*_ > 1) ^33^.

The Freundlich empirical equation is employed to describe heterogeneous systems. It elucidates physical adsorption on surfaces (homogenous and heterogeneous). A non-linear form of the Freundlich equation is expressed as^34^:

5 $${Q}_{m}={K}_{F}\times {Ce}^{(\frac{1}{n})}$$

where *Q*_*m*_ is the amount of metal ion adsorbed (mg kg^−1^), *K*_*F*_ is the Freundlich constant representing the adsorption capacity (mg g^−1^), *C*_*e*_ is metal equilibrium concentration (mg L^−1^), and the adsorption intensity was expressed by *n* as a constant value (dimensionless)^34^.

Temkin isotherm model assumes that the adsorption energy decreases linearly with the surface coverage due to adsorbent–adsorbate interactions. The linear isotherm equation is expressed as^18^:

6 $${Q}_{m}=A+{K}_{T}\times Ln({C}_{e})$$

where *Q*_*m*_ and C_e_ are the same as above mentioned, *A* is the constant and intercept of equation (Lg^−1^), and *K*_*T*_ is the constant value of the Temkin equation that is represented the sorption heat (J mol^−1^)^35^. The value of heat of adsorption (*K*_*T*_) less than 40 kJ mol^−1^ indicates a physical adsorption and more than 40 kJ mol^−1^ represents chemical adsorption^36^.

The Dubinin–Radushkevich (D–R) isotherm is more general than the Langmuir isotherm because it does not assume a homogeneous surface or constant adsorption potential^9^. It was applied to distinguish between the physical and chemical adsorption of Cu(II) ions^9^. The non-linear form of (D–R) isotherm equation is expressed as^37^:

7 $${Q}_{m}={q}_{DR}\mathit{exp}({-\beta }_{DR }\times {\varepsilon }_{DR }^{2})$$

where Q_m_ is the adsorbed ion amount per unit weight (mmol g^−1^), *q*_*DR*_ (mmol g^−1^) and *β*_*DR*_ (mol^2^ J^-2^) are the empirical constants of the equation and *ε*_*DR*_ is related to the Polanyi potential that expressed as RT *ln* (1 + (1/C_e_)), where *R* and *T* are the gas constant (8.314 J mol^−1^ K^−1^) and absolute temperature (K) respectively^37^. The adsorption free energy (*E*) generally is related to the value of β_DR_ that can be computed from the following equation^37^:

8 $$E=1/\sqrt{{2\beta }_{DR}}$$

The type of adsorption mechanism is related to adsorption free energy (kJ mol^−1^)^37^. Physisorption, ion exchange and, chemisorption mechanisms have adsorption energy in the range of 1–8 kJ mol^−1^, 8–16 kJ mol^−1^, and 20–40 kJ mol^−1^ respectively^37^.

### Background electrolyte and ionic strength

Different concentrations (0.01, 0.1, and 0.5 M) of chloride salts (CaCl_2_ and NaCl) were added to 5 mL of Cu(II) solutions (0, 2.5, 5, 10, 20, 40, and 80 mg L^−1^) with 0.1 g nanoparticles. The pH of each sample was fixed at 7.0 and monitored at the end of each experiment to verify any changes which were statistically negligible. The suspensions were shaken for 2 h and left overnight for equilibration^38^. Equilibrium Cu(II) concentration in solution samples were measured by Flame Atomic Absorption Spectrophotometer, after centrifuging at 10,000 rpm for 30 min.

### Thermodynamics

In any adsorption procedure, values of thermodynamic parameters such as Gibb’s free energy (ΔG°), standard enthalpy (ΔH^0^) and entropy (ΔS^0^) must be taken into consideration in order to determine the thermodynamic nature of a process^18^. Values of thermodynamic parameters are the actual indicators for practical application of a process^26^. Adsorption of Cu(II) onto nanoadsorbents base on thermodynamic studies was calculated at a range of temperature (298, 303, 308, and 313 K). The Gibb’s free energy (ΔG°) was calculated by the following equation^18^.

9 $${\Delta G}^{0}=-RT ln{(K}_{c})$$

where *R*, *T* and *K*_*c*_ are commonly gas constant (8.314 J mol^−1^ K^−1^), absolute temperature (K), and the equilibrium adsorption constant, respectively^11^. The Arrhenius equation was used to determine kinetic of adsorption reactions. It can be expressed as the following equation^11^:

10 $$K\left(T\right)={(K}_{B}\times T /h \times {C}_{0}{) exp}^{-{\Delta G}^{0}/RT}$$

where *K*_*B*_ is the Boltzmann constant (1.380 × 10^–23^ J K^−1^), *T* is the absolute temperature (K), *h* is the plank constant (6.626 × 10^–34^ J S^−1^), *C*_*0*_ is the concentration (mol L^−1^), *R* is the gas constant (8.314 J mol^−1^ K^−1^), and ΔG° is Gibb’s free energy (KJ mol^−1^) respectively^11^.

Standard enthalpy (ΔH^0^) and entropy (ΔS^0^) were determined from the Van’t Hoff isotherm equation as follow^11^:

$$\mathrm{ln}K=\frac{{\Delta S}^{0}}{R}-\frac{{\Delta H}^{0}}{RT}$$

ΔH° and ΔS° were obtained from the slope and intercept of the plot of ln K vs 1/T.

### Statistics

The coefficient of determination (R^2^) and the root mean square error (RMSE) statistics were used to evaluate the goodness of fit and absolute error measures respectively. The RMSE is expressed as^39^:

12 $$RMSE= \sqrt{\sum_{i=1}^{n}{({P}_{i}-{O}_{i})}^{2}/n}$$

where *P*_*i*_ and *O*_*i*_ are the predicted and measured values of Cu(II) concentrations sorbed to nanoadsorbents and *n* is the number of initial Cu(II) concentrations applied in sorption experiment respectively^30^. Root mean square error (0 to + ∞) was used as an index of absolute error. A lower RMSE and higher R^2^ values show better goodness of fit between measured and estimated data^30^. Statistical evaluation was performed using statistical analysis software (SAS 9.4; Institute, 2011), speciation of Cu(II) in aqueous was accomplished by Visual MINTEQ 3.1, and bar chart, and line graphs were drawn using Microsoft Office Excel 2015 software. Optimization of various parameters of adsorption models was performed using Solver 2015.

### Complying with relevant institutional, national, and international guidelines and legislation

The authors declare that all relevant institutional, national, and international guidelines and legislation were respected.

## Results

### Characterization

**Figure 1:**
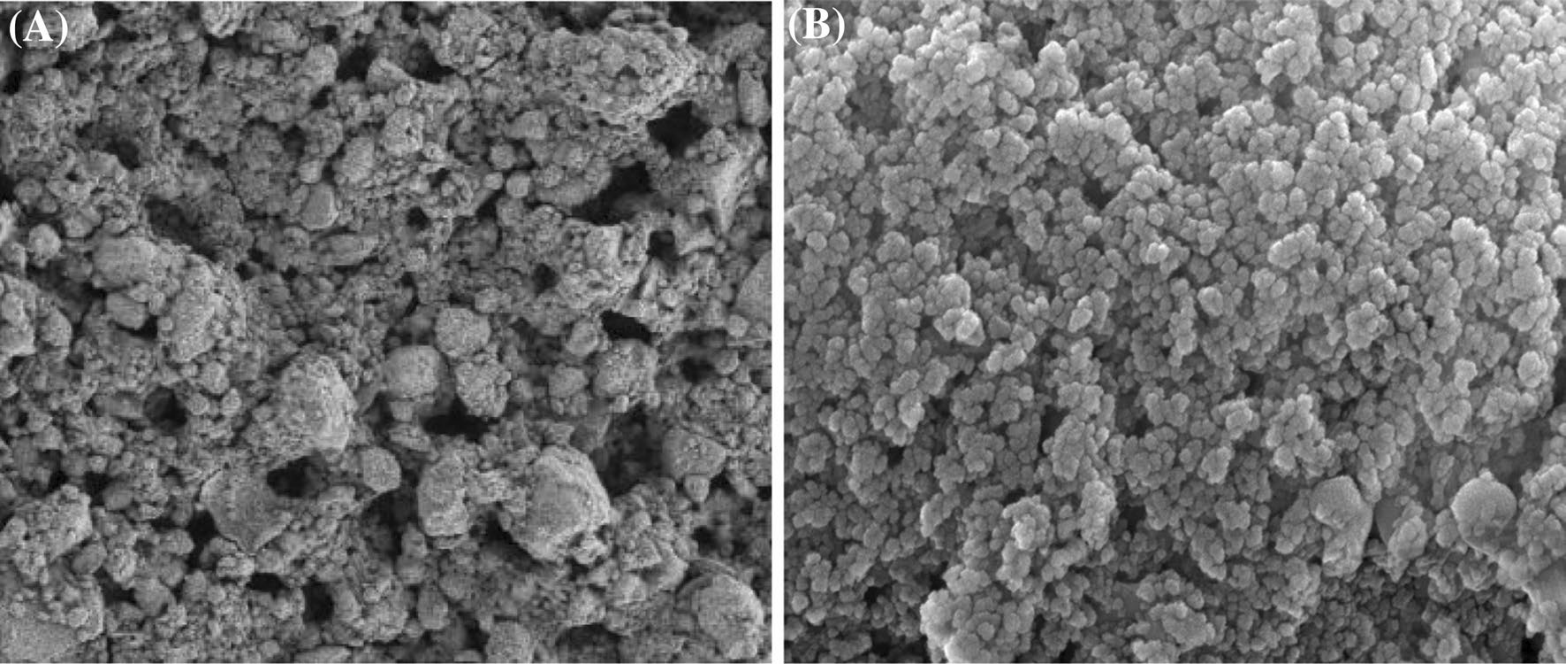
SEM images of **(A)** TiO_2_, **(B)** Al_2_O_3_ nanoparticles.

**Figure 2:**
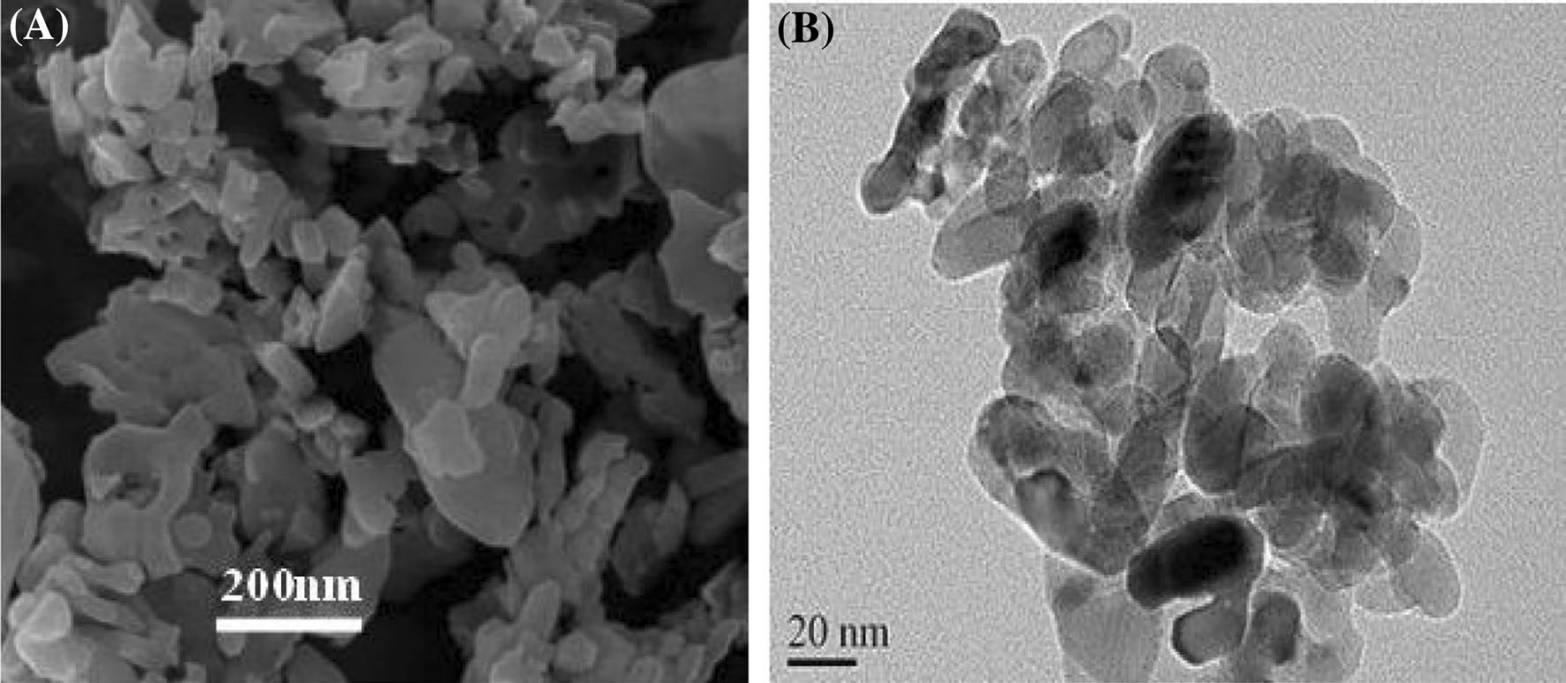
TEM images of **(A)** TiO_2_, **(B)** Al_2_O_3_ nanoparticles.

The morphological features and crystal structure of the nanoparticles obtained from SEM–EDX analysis is provided in Fig. 1. The nanoparticles showed a rough sphere-like structure before adsorption. Although γ-Al_2_O_3_ nanosorbents showed a high surface homogeneity, TiO_2_ nanoparticles had an irregular structure, thus makes possible the adsorption of Cu(II) ions on different parts of the adsorbent (Fig. 1). The mean diameters of TiO_2_ and γ-Al_2_O_3_ nanoparticles were 20 nm and 200 nm respectively based on TEM analysis (Fig. 2).

Energy dispersive X-ray spectroscopy (EDX) analysis was used to characterize the elemental composition of the nanoTiO_2_ and γ-Al_2_O_3_. The results are summarized in Table 1. It can be found from the EDX analysis that titanium (Ti) and oxygen (O) are the main elements presented in the nano-TiO_2_, which confirms the formation of TiO_2_. The EDX data shows that other elements percentage were very negligible in TiO_2_ nanoparticles. Same results were found in γ-Al_2_O_3_ nanoparticles (Table 1), which mainly consists of aluminium (Al) and O. The molecular ratio of Ti: O (nano TiO_2_) and Al: O (nano γ-Al_2_O_3_) of the nanoparticles, calculated from EDX and quantitative analysis data, is close to that of bulk, which again confirmed that the grown nanoparticles are pure.

**Table 1:** Elemental composition based on energy dispersive X-ray spectroscopy (EDX) analysis of nanoTiO_2_ and γ-Al_2_O_3_.

Titanium oxide nanopowder (TiO_2_)—%												
TiO_2_-rutile	Al	Ca	Co	Cr	Fe	K + Na	Mo	Mg	P	S	Si	W
≥ 99.9	≤ 0.003	≤ 0.005	≤ 0.01	≤ 0.005	≤ 0.005	≤ 0.005	≤ 0.005	≤ 0.01	≤ 0.01	≤ 0.005	≤ 0.003	≤ 0.01

Aluminum oxide nanoparticles (gamma) certificate of analysis—wt.%												
Al_2_O_3_	Ca	Fe	Mg	Na	Si	Cr	Mn	Co				
≥ 99.9%	≤ 0.02	≤ 0.01	≤ 0.03	≤ 0.02	≤ 0.02	≤ 0.04	≤ 0.03	≤ 0.02				

**Figure 3:**
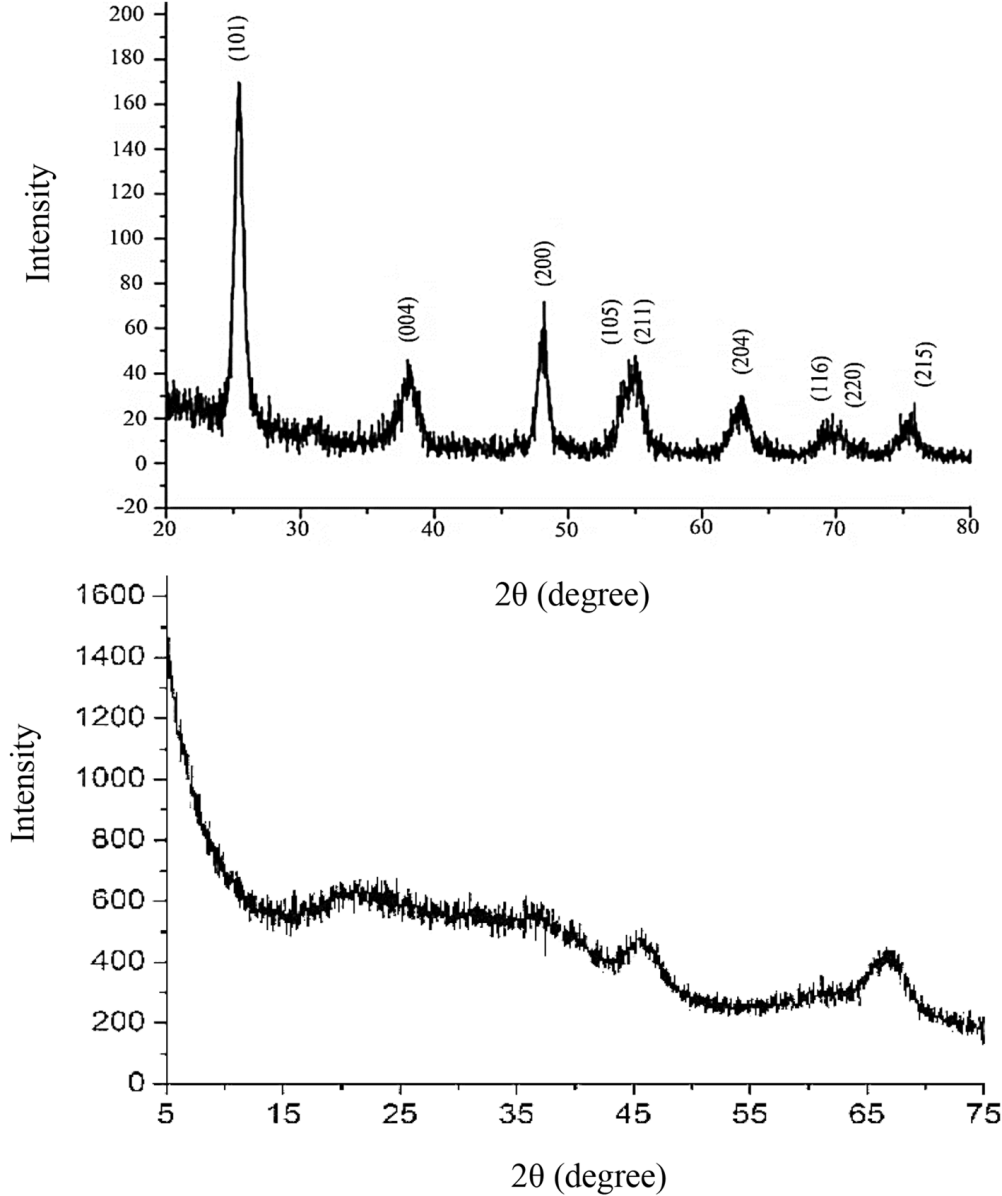
The XRD pattern of **(A)** TiO_2_ and **(B)** γ-Al_2_O_3_ nanoparticles.

Structural information of the final product could be given by powder X-ray diffraction (XRD) analysis. In the XRD pattern of sample (Fig. 3), all the observed peaks can be indexed to a pure tetragonal anatase phase (JCPDS card, 21-1272) and aluminum oxide. No peak of other phases was observed, which indicates that the products are pure and well crystallized.

### Speciation

**Figure 4:**
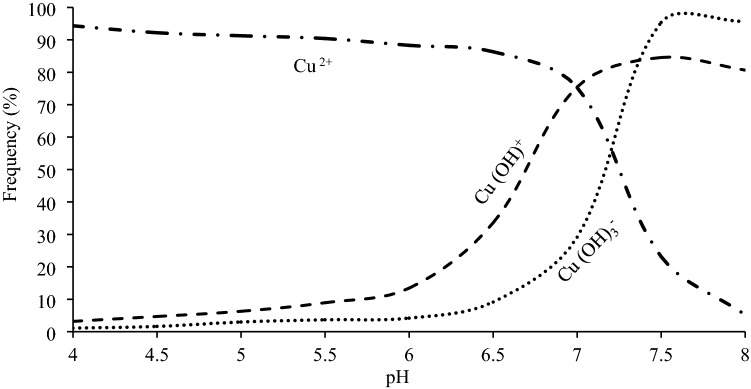
Copper speciation in equilibrium solution using visual MINTEQ (ionic strength 0.01 M CaCl_2_).

**Figure 5:**
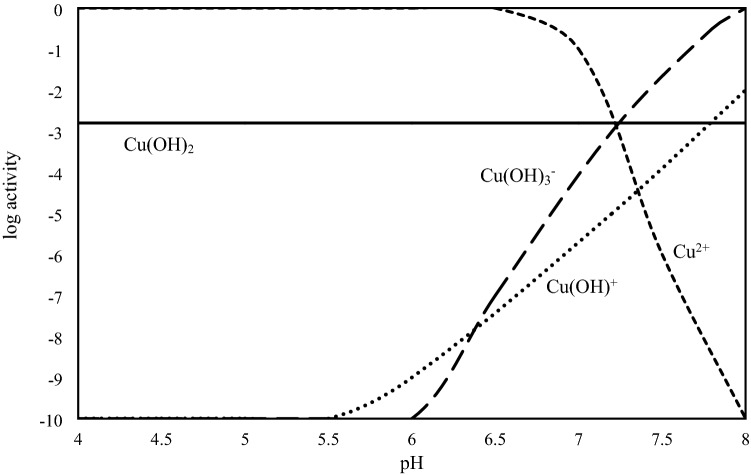
The pa-pH diagram for cupric solubility in the equilibrium solution using visual MINTEQ (ionic strength 0.01 M CaCl_2_).

The frequency of various chemical forms of Cu in aqueous solution at different pH values with the nanosorbents is shown in Fig. 4. Free metal ions were predominant form in solutions with two nanoparticles at pH values ranging from 4.0 to 6.5, however the proportion of other Cu species were negligible at the pH. The frequency of Cu(OH)^+^ and Cu(OH)_3_^-^ increased with increasing of pH above 7.0. The results are in agree with their activity at various pH values (Fig. 5).

Saturation indices of different Cu(II) minerals were all below zero and undersaturated in solutions with TiO_2_ and γ-Al_2_O_3_ nanoparticles at various pH values (Table 2).

**Table 2:** Saturation indices of Cu minerals in equilibrium solution at different pH values using visual MINTEQ (ionic strength 0.01 M CaCl_2_)**.**

Adsorbent	Cu minerals	pH 4	pH 6	pH 8
TiO_2_	Cu (OH)_2_	− 15.28	− 9.28	− 6.20
	Cu_4_Cl_2_ (OH)_6_	− 12.36	−10.29	− 8.13
	Cu_2_O	− 11.55	− 9.55	− 8.29
	Cu_2_CO_3_ (OH)_2_	− 20.37	− 10.62	−5. 25
	Azurite	− 25.14	− 12.20	− 6.62

		pH 6	pH 7	pH 8
γ-Al_2_O_3_	Cu (OH)_2_	− 15.30	− 11.25	− 7.12
	Cu (OH)_3_	− 7.25	− 4.32	− 2.68
	Cu_4_Cl_2_ (OH)_6_	− 10.13	− 8.25	− 7.13
	Cu_2_O	− 13.28	− 12.36	− 8.28
	Cu_2_CO_3_ (OH)_2_ Azurite	− 9.32 − 11.27	− 7.28 − 9.65	− 5.70 − 5.14

### Effect of pH on adsorption

**Figure 6:**
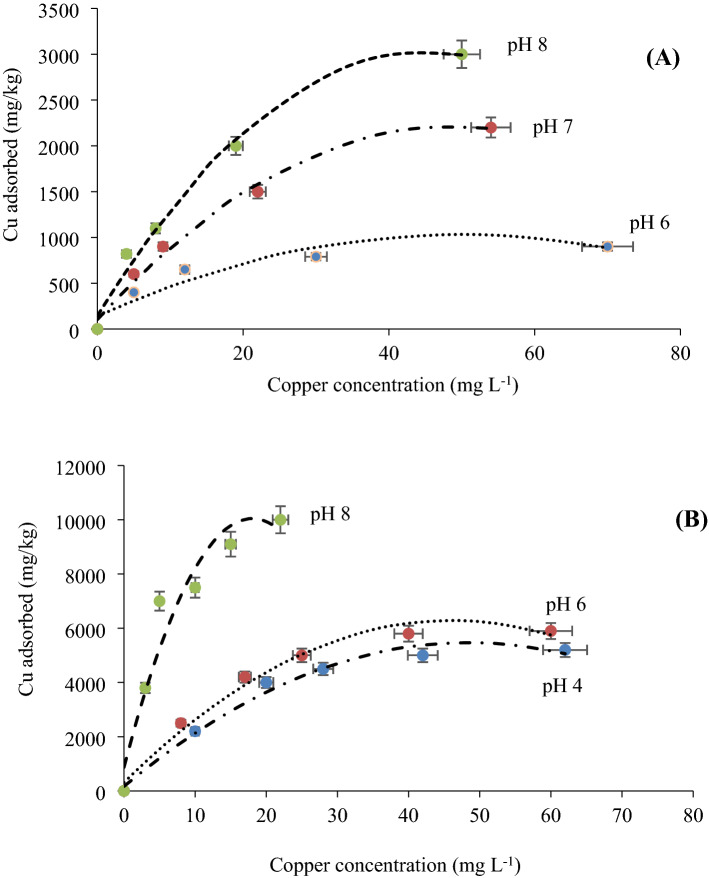
Effect of pH on Cu(II) adsorption by **(A)** γ-Al_2_O_3_, and **(B)** TiO_2_ nanoparticles.

pH is an essential parameter of adsorption study which affects the sorption capacity of the nano-adsorbents and regulates the feasibility of treatment method. The effect of pH on Cu(II) adsorption on the nanoparticles is shown in Fig. 6. The results indicate that the TiO_2_ nanoadsorbents have a greater adsorption capacity than γ-Al_2_O_3_ for all pH values from 4.0 to 8.0. Copper (Cu(II)) adsorption significantly increased by increasing of pH values for both nanoparticles (Fig. 6).

Similarly, according to Li et al.^40^, Cu(II) reached its highest adsorption on functionalized bentonite at pH > 6.5. This adsorption exhibited a dependence on the electrostatic interactions related to the presence of functional groups. The heavy metals adsorption, as determined in several studies, is favored at moderate pH values than at lower pH values. For instance, nanofiber chitosan/TiO_2_ chelated Cu(II) ions with minimum removal at 2–4 pH values and maximum removal at more than 6 pH values^31^.

Adsorption is a time-dependent processes. The adsorption kinetics of Cu(II) with the adsorbents occurred rapidly and reached the equilibrium after 4 h (data not shown) in this study. Therefore, a contact time of 4 h was selected for all batch adsorption experiments in order to ensure that the equilibrium was established. Although the kinetic study was not reported, the results of previous researches of Cu(II) adsorption kinetics on TiO_2_ and γ-Al_2_O_3_ nanoparticles demonstrated that the Cu(II) adsorption by TiO_2_ occurred rapidly and reaching adsorption equilibrium after 5 h and the pseudo- second order equation described the kinetic data well as the predominant mechanism^41^. Meanwhile previous researches^42^ demonstrated that adsorption kinetics were the best fitting by a pseudo-second order kinetic model. This model is more likely to predict the kinetic behavior of sorption, with chemical sorption being the rate-determining step. Previous researches^43^ reported that the pseudo- second order kinetic equation, which relies on the assumption that the chemical reaction might be considered as the rate-controlling step, is the better model in the studied adsorption systems. In this process, the metal ions join the adsorbent surface by forming a chemical bond through sharing or exchange of electrons and tend to find sites that maximize their coordination number with the surface. Generally, a continuous multi-step process may be taking place during the sorption of a sorbate by a porous sorbent.

To determine the maximum Cu(II) adsorption and adsorption parameters, four equilibrium models were fitted to the experimental data, including the Freundlich, Langmuir, Temkin, and Dubinin Radushkevich equations. Table 3 represents the calculated values of isotherm parameters. Comparing of the statistical parameters listed in Tables 3 and 4, indicated that the Freundlich isotherm was the best to predict the equilibrium adsorption behavior. This indicated the surface heterogeneity of nanoparticles, uniform energy distribution, and reversible Cu(II) adsorption during the sorption process. The maximum adsorption capacities (S_m_) and the Langmuir equation constant parameter (K_L_) were equal to 9288 and 3607 mg kg^−1^ and 0.78 and 0.11 L mg^−1^ for Cu(II) adsorption on TiO_2_ and γ-Al_2_O_3_ nanoparticles at the highest pH value respectively, which decreased by decreasing of pH (Table 3). The adsorption capacity of TiO_2_ nanoparticles were 2.5 times more than γ-Al_2_O_3_, which indicated TiO_2_ nanosorbents were more efficient for Cu(II) removal than γ-Al_2_O_3_ nanoparticles. Comparing of the results with various previous studies shows that experimental data of the present study was found to be lower than some recently reported studies^26^. The higher values of K_L_ confirmed the ease of adsorption processes^44, 45^. All the values of R_L_ lie between 0.09 and 0.30 for all studied pH values, indicating favorable adsorption of Cu (II) onto the nanoparticles (R_L_ < 1).

**Table 3:** Calculated isotherm equation parameters for adsorption of Cu(II) by TiO_2_ and γ-Al_2_O_3_ nanoparticles.

Adsorbent	pH	Freundlich				Langmuir					Temkin				Dubinin–Radushkevich				
		K_F_ (L mg^−1^)	n	R^2^	RMSE	S_m_ (mg kg^−1^)	K_L_ (Lmg^−1^)	R_L_	R^2^	RMSE	A (L g^−1^)	K_T_ (J mol^−1^)	R^2^	RMSE	q_DR_ (mmol g^−1^)	β_DR_ (mol^2^ J ^−2^)	E (kJ mol^−1^)	R^2^	RMSE
TiO_2_	4	1640	3.22	0.90	0.04	7371	0.07	0.30	0.89	0.11	2000	890	0.89	0.23	0.003	3.14 × 10^–5^	1.95	0.91	0.09
	6	1750	3.03	0.92	0.05	7750	0.09	0.19	0.90	0.14	2000	1013	0.82	0.19	0.021	6.09 × 10^− 4^	3.26	0.90	0.05
	8	3885	2.94	0.94	0.06	9288	0.78	0.10	0.91	0.13	4438	1621	0.91	0.24	0.023	6.84 × 10 ^−6^	5.14	0.92	0.09
γ-Al_2_O_3_	6	374	3.71	0.95	0.01	1132	0.04	0.21	0.96	0.12	381	179	0.90	0.16	0.001	2.05 × 10 ^−5^	1.44	0.93	0.06
	7	415	2.50	0.99	0.05	3070	0.08	0.14	0.98	0.15	352	500	0.94	0.13	0.001	3.19 × 10 ^−5^	1.32	0.91	0.08
	8	660	2.50	0.98	0.06	3607	0.11	0.09	0.97	0.09	176	783	0.95	0.11	0.003	3.55 × 10 ^−5^	1.09	0.94	0.05

*K*_*F*_ Freundlich equation constant; *n* empirical constant of the Freundlich equation; *S*_*m*_ maximum adsorption capacity; *K*_*L*_ Langmuir equation constant; *A* empirical constant; *K*_*T*_ constant value of the Temkin equation; *q*_*DR*_* and β*_*DR*_ empirical Dubinin–Radushkevich equation constants, *E* adsorption free energy.

**Table 4:** Copper adsorption capacity by TiO_2_ and γ-Al_2_O_3_ nanoparticles at different pH values.

Adsorbent	pH	Freundlich		Langmuir		Temkin		Dubinin–Radushkevich	
		Q_me_ (mg kg^−1^)	Q_mo_ (mg kg^−1^)	Q_me_ (mg kg^−1^)	Q_mo_ (mg kg^−1^)	Q_me_ (mg kg^−1^)	Q_mo_ (mg kg^−1^)	Q_me_ (mg kg^−1^)	Q_mo_ (mg kg^−1^)
TiO_2_	4	5200	5630	5200	5320	5200	5120	5200	5140
	6	5900	6100	5900	6300	5900	5720	5900	6010
	8	10,000	10,240	10,000	10,500	10,000	10,060	10,000	9126
γ-Al_2_O_3_	6	900	960	900	1200	900	1110	900	980
	7	2200	2320	2200	2360	2200	2160	2200	1820
	8	3000	3128	3000	3230	3000	3030	3000	3050

Q_me_ and Q_mo_ are the maximum Cu(II) concentration and predicted by various isotherm models respectively.

### Removal efficiency

**Figure 7:**
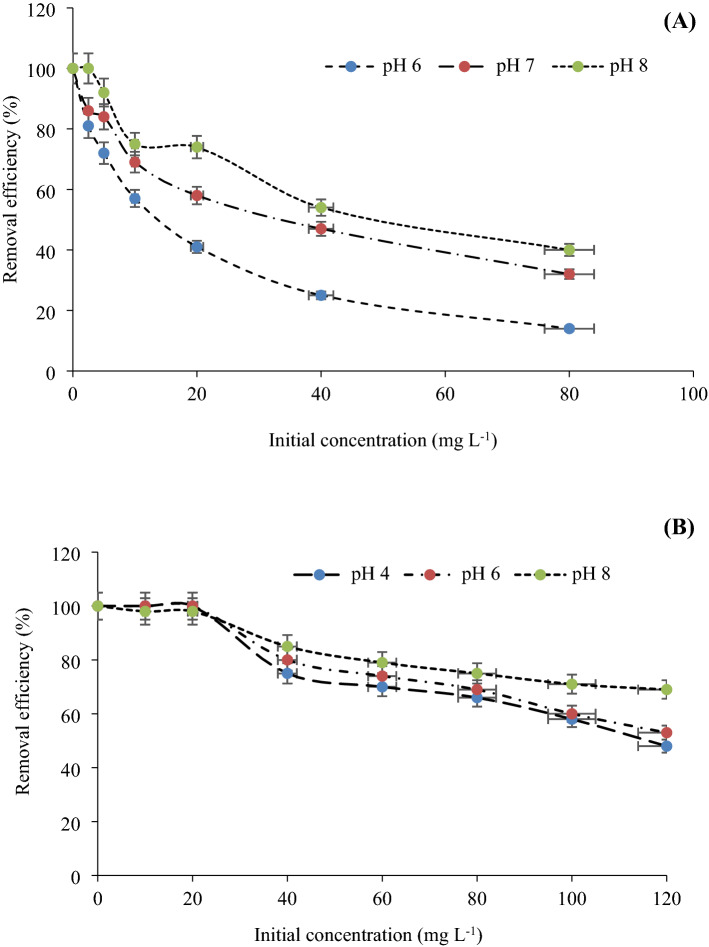
Effect of initial concentration and pH on Cu(II) removal efficiency by **(A)** Al_2_O_3_, and **(B)** TiO_2_ nanoparticles.

Initial metal concentration is one of the most important parameters for the removal of metal ions from aqueous solutions. The effects of different Cu(II) initial concentrations on the removal efficiency of the adsorbents at different pH values are shown in Fig. 7. The Cu(II) removal percentage with TiO_2_ nanoparticles was decreased from 98 to 90% as the Cu(II) initial concentration increased with a maximum of 80 mg L^−1^. The removal efficiency kept on decreasing and reached a constant value with increasing of initial concentration. Same trend was found with γ-Al_2_O_3_ nanoparticles. Hence, initial concentrations of 10 and 5 mg L^−1^ were taken as the maximum value for Cu(II) removal with TiO_2_ and γ-Al_2_O_3_ nanoparticles respectively (Fig. 7). From Fig. 7a it seems that at higher concentration the removal efficiency suddenly decreases. It was clearly interpreted that desorption occurs at higher concentration. This means the adsorbent is not stable and was not feasible for further usage.

### Ionic strength and background electrolyte

Different adsorbates compete for adsorption sites characterized by maximum heat of adsorption and minimum free energy of adsorption^46^. The competitive effect of some ions (Ca^2+^ and Na^+^) on Cu(II) adsorption with TiO_2_ and γ-Al_2_O_3_ nanoparticles was investigated in the presence of CaCl_2_ and NaCl as various background electrolytes at different ionic strengths (0.01, 0.1, and 0.5 M) (Table 5). Ranging of 0.01 to 0.5 M ionic strength caused to decrease of Cu(II) adsorption with TiO_2_ nanoparticles. More decreasing of Cu(II) adsorption was found in the presence of CaCl_2_ than NaCl, which may be due to the higher competition of Ca^2+^ ions with Cu(II) for adsorption on active surface sites^44^. The results of previous researches^7^ showed the significant decrease of Cu(II) adsorption using various nanoparticles by increasing of Ca^2+^ concentration in solution. For γ-Al_2_O_3_, the behavior was similar, however, more Cu(II) adsorption was found with TiO_2_ than γ-Al_2_O_3_ nanosorbents in the constant ionic strength and initial concentration (Table 5).

**Table 5:** Copper adsorption (mg kg^−1^) in the presence of NaCl and CaCl_2_ as background electrolytes at various ionic strengths.

Adsorbent	Initial Cu (II) concentration	NaCl				CaCl_2_		
		Ionic strength (M)				Ionic strength (M)		
		0.01		0.1	0.5	0.01	0.1	0.5
TiO_2_	10	907		907	907	508	507	507
	20	1531		1531	1530	962	960	959
	40	2810		2810	2805	2310	2307	2307
	60	3707		3685	3665	2992	2992	2991
	80	4510		4510	4505	3885	3885	3425
γ-Al_2_O_3_	2.5	99		98	95	53	52	47
	5	235		232	230	175	145	135
	10	455		450	450	270	270	265
	20	1100		1095	1900	835	820	812
	40	2200		2190	2180	1825	1825	1820
	80	2875		2825	2800	2125	2125	2115

### Thermodynamic

The thermodynamic parameters were determined for feasibility and spontaneity of the adsorption process at different pH values. The Gibbs’s free energy (ΔG^0^) and ΔH° and ΔS° have been calculated using thermodynamic relationship (Table 6).

**Table 6:** Calculated thermodynamic parameters of Cu(II) adsorption on the nanoparticles at different pH values.

Adsorbent	pH values	ΔG°_298_ (KJ mol^−1^)	ΔG°_303_ (KJ mol^−1^)	ΔG°_308_ (KJ mol^−1^)	ΔG°_313_ (KJ mol^−1^)	ΔH° (KJ mol^−1^)	ΔS° (KJ mol^−1^ K^−1^)	R^2^
TiO_2_	4	−15.36	−21.36	−29.68	−33.55	−68.15	−200.53	0.89
	6	−16.28	−29.16	−35.65	−39.24	−71.25	−215.22	0.81
	8	−17.93	−32.19	−38.19	−45.69	−73.29	−217.62	0.87
γ-Al_2_O_3_	6	−17.65	−33.98	−39.95	−50.16	−76.58	−221.53	0.91
	7	−18.34	−37.98	−43.22	−53.29	−81.42	−224.23	0.86
	8	−18.92	−40.39	−48.26	−57.35	−84.39	−227.41	0.93

## Discussion

Based on the International Union of Pure and Applied Chemistry (IUPAC), the TiO_2_ and γ-Al_2_O_3_ nanoparticles consists of micropores with diameter less than 20˚ A. The morphological properties of the nanoparticles surface are suitable for adsorption processes. The specific surface area was measured by BET equation. The surface area of TiO_2_ nanoparticles (200 m^2^ g^−1^) was more than γ-Al_2_O_3_ (150 m^2^ g^−1^), which can influence the adsorption capacity of the nanomaterials^47^. Previous researchers demonstrated that increasing of surface area and decreasing of nanoparticles diameter caused to increase of adsorption capacity^27^.

Several factors can influence the chemical behavior, biotoxicity, and bioavailability, and ultimately chemical fate of either nutrients or heavy metals in the environment^48^. Solution pH is a critical factor that affect the distribution of absorbable species of heavy metals^30^. Besides, the adsorption mechanism (surface precipitation vs. adsorption) can affected by pH. Based on the obtained results, no precipitation was found in solution with the nanoparticles (Table 2) and the physical adsorption on nanoparticle was accrue. It can be inferred that the increase of pH results in less H^+^ available to compete with Cu(II) and/or Cu(OH)^+^ for the same adsorption sites on the surface of the adsorbent. Furthermore, as the pH increases, Cu(II) will hydrolyze to Cu(OH)^+^, which is the species most readily adsorbed^49^. Increasing of Cu(II) adsorption with nano-oxides was attributed to the pH-dependent charge of these adsorbents^4^. Meanwhile, electrostatic repulsion between positively charge Cu(II) ions and positively nanoparticle surfaces generated at the pH less than pH_ZPC_, causes to diminish the adsorption of Cu ions as outer sphere complexes^50^. However, inner sphere complexes are responsible for metal adsorption at pH > pH_ZPC_. As can be seen in Fig. 6. b, more Cu(II) adsorption on TiO_2_ nanoadsorbents was occurred at pH values above 6.0 as the pH_ZPC_ of the TiO_2_ nanoparticles, due to deprotonation of hydroxyl groups on nanoparticle surface and increase of electrostatic forces between Cu(II) ions and negatively surface charges^30^. Decreasing of Cu(II) adsorption at pH 4.0 is due to electrostatic repulsive force between Cu(II) ions and positively surface charge of the nanoadsorbent, resulted from protonation of surface functional groups. Though, at low pH the protonated active sites numbers increase and caused a great repulsion with positive charged toxic pollutants that greatly reduces the adsorption capacity of the nanoadsorbent^31^. At very high pH values, several complexes between metal species and OH groups formed that blocked the large numbers of adsorbent active sites and reduced their adsorption capacity^51^. Previous researches showed that Cu(II) adsorption on γ-Fe_2_O_3_ nanoparticles was maximum at pH 7 value^52^. Same result was found by Huang et al.^53^, who found that the Cu(II) adsorption reached its highest at pH values more than 6.5.

Although the same adsorption trend was obtained with both nanoparticles, the adsorption capacity of TiO_2_ was more than γ-Al_2_O_3._ Previous researches demonstrated the high adsorption capacity of TiO_2_ for removal of heavy metals from aqueous solutions^27^. As shown in Fig. 6.a, The Cu adsorption was reached to the maximum value at pH values more than 7.2 (pH_ZPC,_) ^1^. The most abundant Cu species in solution were Cu(OH)_2_ and Cu(OH)^+^ at pH above 6.0 (Fig. 4). Same results were reported by other researchers^8^.

The values of n parameter from the Freundlich isotherm for the adsorption of Cu(II) by TiO_2_ and γ-Al_2_O_3_ nanoparticles were all greater than 1 at various pH values, indicating Cu(II) ions adsorption on the adsorbent surface were favorable^3^. The correlation coefficient of the Temkin isotherm equation is small, which indicates that the adsorption process of Cu(II) by the nanoparticles is not suitable for description by the Temkin isotherm model (Table 3). The Dubinin Radushkevich equation was fitted to specify the chemical or physical adsorption mechanisms. The adsorption free energy of the Dubinin Radushkevich equation was less than 8 kJ mol^−1^ with both nanoparticles at different pH which is evidence of physically adsorption mechanism of Cu(II) onto nanoparticle surface (Table 3). In physical adsorption, the individuality of the adsorbate and the adsorbent are preserved. In return, chemisorption occurs as a chemical reaction between the adsorbate and the surface. Also, new chemical bonds are generated at the adsorbent surface^35^.

At the initial stage of the adsorption process and low Cu(II) concentration, there were free surface adsorption sites on nanoparticles which can increase the adsorption processes and removal efficiency. However, the adsorption rate was decreased by increasing of the Cu(II) concentration and occupation of active adsorption sites^47^. The adsorption at different concentrations is rapid in the initial stages and gradually decreases during the progress of adsorption until the equilibrium is reached^30^. The high adsorption rate at the beginning was due to the adsorption of copper ions by the exterior surface of the adsorbent. When saturation was reached at the exterior surface, the metal ions entered the pores of adsorbent and were adsorbed by the interior surface of the particles^9^. The initial faster rates of adsorption may also be attributed to the presence of large number of binding sites for adsorption and the slower adsorption rates at the end is due to the saturation of the binding sites and attainment of equilibrium^31^. Based on Van Tran et al. (2020), the adsorbent surface is saturated at higher levels of initial concentration, which is attributable to enhanced affinity of the interactions between molecules and adsorption sites on adsorbent surface until reaching a saturation threshold. Previous researches showed that the initial metal concentration have important role for mass transfer between the aqueous and solid phases^37^. In order to environmental remediation, the optimum initial concentration of γ-Al_2_O_3_ and TiO_2_ were reported as 8 mg L^−1^ and 15 mg L^−1^ in previous researches^10^.

In water, salt is present in a wide range of concentrations depending on the source and the quality of the water^22^. The presence of salt leads to high ionic strength, which may significantly affect the performance of the adsorption process. The reason for this is that Ca^2+^ and Na^+^ ions in the aqueous phase compete effectively with positively charged Cu(II) ions for the same binding sites on the adsorbent surface^27^. Additionally, salt screens the electrostatic interaction between adsorbent and adsorbate and the great ionic strength influences on the activity coefficient of Cu(II), which should decrease the adsorbed amount with the increase in salt concentration^51^. Same results were reported by Van Tran et al. (2020), who found that the ionic strength and background electrolyte affected the adsorption competition.

The results of previous studies on Cu(II) adsorption using different nanoparticles are presented in Table 7. Comparison of the obtained results with previous studies shows that more Cu(II) concentration was adsorbed with TiO_2_ and γ-Al_2_O_3_ nanoparticles (the present study) than others (Table 7). Meanwhile, the results show that the equilibrium in the process of Cu(II) adsorption by TiO_2_ and γ-Al_2_O_3_ nanoparticles (the present study) was obtained earlier than other adsorbents.

**Table 7:** Copper (Cu(II)) adsorption with different adsorbents.

Adsorbent	Initial metal concentration (mg L^−1^)	pH	Temperature (K)	Contact time (min)	Maximum adsorption (mg kg^−1^)	Reference
Nano TiO_2_	80	8.0	298	240	10,000	This study
Nano γ-Al_2_O_3_	80	8.0	298	240	3000	This study
Nano γ-MnOOH	25	7.5	298	120	1100.92	^4^
Polyethyleneimine modified wheat straw	500	7.5	293	240	4800.60	^22^
Nano γ-Fe_2_O_3_	80	7.0	298	120	600.81	^52^
Powdered activated carbon (PAC)	60	7.0	298	130	2300.61	^54^
Magnetite nanoparticles (Fe_3_O_4_)	60	7.0	298	150	1300.37	^54^
Fe_3_O_4_–MnO_2_–EDTA composite	100	6.0	298	60	2500.72	^55^
ZnO nanoparticles	100	> 6.0	298	150	2000.42	^56^

The spontaneous nature of the adsorption processes can be determine by negative values of Gibbs free energy (ΔG°), means that no energy input from outside of the system is required^6^ and the exothermic performance of the adsorbed and the adsorbent interaction. If the ΔG° values ranged from − 20 to 0 kJ mol^−1^, it means the physical adsorption process^24^. The more negative values of ΔG° imply a greater driving force to the adsorption process, and confirming that the adsorption of Cu(II) onto the nanoparticles is spontaneous and thermodynamically favorable. According to the results, increase of pH caused to increase of the kinetic of adsorption reactions. The spontaneous nature was slowly found at the lowest pH than the highest. The values of ΔH° are negative, indicating that the adsorption process is exothermic in nature. The negative values of ΔS° indicate greater order of reaction during adsorption of Cu(II) on the nanoparticle surface.

## Conclusion

In general, the results of the present study showed that TiO_2_ and γ-Al_2_O_3_ nanoparticles, especially TiO_2,_ had the high capacity for Cu(II) removal from aqueous solution. Speciation data showed that the physical adsorption was the main mechanism for Cu(II) removal. The removal efficiency was increased by increasing pH and initial concentration. The ionic strength had the inverse effect on Cu(II) adsorption, which decreased significantly in the presence of 0.01 M CaCl_2_ background electrolyte. The spontaneous adsorption processes was occur at different pH values. Using of TiO_2_ nanomaterial is an effective method for elimination of Cu(II) polluted environments.
